# Single-staged laparotomy versus multiple-staged laparotomy for traumatic massive hemoperitoneum with hemodynamic instability: a single-center, propensity score-matched analysis

**DOI:** 10.1186/s12893-022-01660-6

**Published:** 2022-06-02

**Authors:** Masaki Matsuda, Makoto Sawano

**Affiliations:** Department of Emergency and Critical Care Medicine, Saitama Medical Center, Saitama Medical University, 1981 Kamoda, Kawagoe-shi, Saitama-ken 350-8550 Japan

**Keywords:** Damage control, Damage control resuscitation, Hemoperitoneum, Propensity score

## Abstract

**Background:**

Currently, damage control surgery (DCS) employing multiple-staged laparotomy (MSL) is a standard hemostatic approach for treating trauma patients with unstable hemodynamics attributable to massive hemoperitoneum. Based on these findings, we had frequently employed MSL as a part of our hemostatic strategy for the patients, but with unsatisfactory outcomes. On the other hand, with the establishment of damage control resuscitation (DCR), it has become possible to avoid trauma-induced coagulopathy and to achieve adequate hemostasis with single-staged laparotomy (SSL). Consequently, our institutional strategy for surgical hemostasis of the patients has gradually shifted from MSL to SSL with implementation of DCR. The purpose of the study is to evaluate the impact of this shift in the strategy by comparing outcomes of the patients between those underwent MSL and those underwent SSL employing propensity score matching.

**Methods:**

This retrospective, single-center, observational study evaluated outcomes of hemodynamically unstable patients with traumatic massive hemoperitoneum requiring surgical intervention between 2005 and 2020. The patient population was divided into two groups: a SSL group and a MSL group. Propensity score matching was used to adjust for differences in baseline characteristics in the two groups, a one-to-one matched analysis using nearest-neighbor matching was performed based on the estimated propensity score of each group. The primary outcome was in-hospital mortality, and secondary outcomes were 48-h mortality and 28-day mortality.

**Results:**

A total of 170 patients met the inclusion criteria; 141 patients underwent SSL, and 29 underwent MSL. In the propensity-matched analysis with 27 pairs, the SSL group had significantly lower in-hospital mortality (odds ratio [OR] 0.154; 95% confidence interval (CI) 0.035 to 0.682) and 28-day mortality (OR 0.200; 95% CI 0.044 to 0.913) than the MSL group, but the 48-h mortality did not differ significantly between the two groups (25.9% vs. 44.4%; OR 0.375; 95% CI 0.099–1.414).

**Conclusions:**

Single-staged laparotomy may be an effective surgical treatment for the traumatic massive hemoperitoneum cases with hemodynamic instability, if conducted following sufficient damage control resuscitation and performed by an experienced surgeon.

**Supplementary Information:**

The online version contains supplementary material available at 10.1186/s12893-022-01660-6.

## Introduction

Currently, damage control surgery (DCS) employing multiple-staged laparotomy (MSL) is a standard hemostatic approach for treating trauma patients with unstable hemodynamics attributable to massive hemoperitoneum. Rotondo et al. reported the usefulness of MSL [1], followed by a series of similar reports [2–6], and some emphasized that expeditious hemostasis was paramount to prevent trauma-induced coagulopathy [7, 8]. Based on these findings, we had frequently employed MSL as a part of our hemostatic strategy for the patients, but with unsatisfactory outcomes.

On the other hand, with the establishment of damage control resuscitation (DCR) including preemptive massive transfusion, limiting crystalloid, and aggressive coagulation factor replacement, it has become possible to achieve adequate hemostasis with single-staged laparotomy (SSL) avoiding trauma-induced coagulopathy [9]. As a result, the surgical approach in our institutional strategy for hemostasis of the patients has gradually shifted from MSL to SSL with implementation of DCR, and SSL has become mandatory since 2012 regardless of hemodynamic status, severity of trauma or coagulation functions.

The purpose of the study is to evaluate the impact of this shift in the strategy by comparing outcomes of the patients between those underwent MSL and those underwent SSL. Prior to 2012, the determination of MSL or SSL was solely dependent on the surgeon’s judgment based on hemodynamics, source of major bleeding, and severity of trauma. Therefore, we employed propensity score matching to exclude as much as possible the interference of these confounding factors on the evaluation of the impact.

## Material and methods

### Study design

The Institutional Review Board of Saitama Medical University approved this study protocol. A retrospective, observational study of patients who underwent urgent laparotomy for massive hemoperitoneum with hemodynamic instability at a level I trauma center in Japan between January 2005 and December 2020 was conducted. From a prospective trauma database maintained by the Department of Emergency and Critical Care Medicine, the data were reviewed to obtain age, sex, mechanism of injury, Glasgow coma scale (GCS) score, systolic blood pressure (SBP), heart rate (HR), Injury Severity Score (ISS), head/neck, chest, abdomen, and extremity Abbreviated Injury Scale (AIS) score, dominant sources of hemorrhage, and surgical procedures.

### Inclusion and exclusion criteria

Trauma patients who underwent surgical intervention because of hemodynamic instability attributable to massive hemoperitoneum were included. Hemodynamic instability was defined as an SBP of 90 mmHg or lower before surgical intervention and massive hemoperitoneum was defined as intra-abdominal hemorrhage that led the surgeons to decide the laparotomy. Patients with prehospital cardiac arrest who could not be resuscitated despite surgical intervention were excluded.

### Operative procedure

The selection of surgical procedure was based on the physicians’ clinical judgment. Based on the surgical management, patients were classified as either undergoing SSL (SSL group) or MSL (MSL group).

### Outcomes

The primary outcome was in-hospital mortality, and the secondary outcomes were 48-h mortality and 28-day mortality.

### Statistical analysis

Patients in the SSL and MSL groups were then matched 1:1 based on their propensity scores, using the nearest neighbor method and a caliper width of 0.2 standard deviations for the propensity score. Age, GCS, SBP, ISS, and dominant sources of hemorrhage (hepatic injury without inferior vena cava [IVC] injury, hepatic injury with IVC injury, great vessel injury, splenic injury, pancreas injury, and mesenteric injury) were selected as potential confounders and entered in the model. In the descriptive statistics, numeric or ordered variables are presented as medians and interquartile ranges, and categorical variables are shown as counts and percentages. Significance was defined as a p value of less than 0.05 or was assessed with 95% confidence intervals in all statistical analyses. Medians and ranges of continuous data were compared using the Mann–Whitney U test. Categorical data were compared using Pearson’s Chi-squared test or Fisher’s exact test as appropriate. In the propensity score-matched analysis, we compared the outcomes between the two groups, using conditional logistic regression analyses. Data were analyzed using SPSS software, version 24 (IBM Corp., Armonk, NY). All data can be referenced in Additional file 1.

## Results

**Fig. 1:**
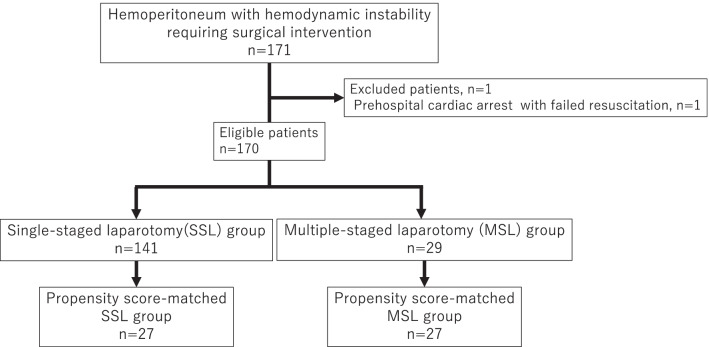
Flow chart of hemodynamically unstable patients with traumatic hemoperitoneum requiring surgical intervention and propensity score matching of patients who underwent SSL and MSL

After the exclusion of ineligible patients, 170 eligible patients were divided into the SSL group (n = 141) and the MSL group (n = 29) (Fig. 1). By one-to-one propensity score matching, 27 pairs from the SSL and MSL groups were selected. The C-statistic for goodness of fit was 0.810 in the propensity score model.

Table 1 shows the demographics and Table 2 shows the dominant sources of hemorrhage of all patients (n = 170) and of the propensity score-matched patients (n = 54). Table 3 shows the surgical procedures in the SSL group. In all patients, the median GCS score, ISS, the proportion of patients with a high AIS (> 3) of the chest and extremities, hepatic injury with IVC injury, splenic injury, great vessel injury, and mesenteric injury were significantly different between the two groups. After propensity score matching, patient distributions were closely balanced between the SSL and MSL groups.

**Table 1:** Demographic characteristics of the SSL and MSL groups

	Before matching			After matching		
	SSL group	MSL group	p value	SSL group	MSL group	p value
Patients, n	141	29		27	27	
Age, y, median (IQR)	44 (22–61)	51 (30–68)	0.224	49 (25.5–63)	49 (27.5–65.5)	0.797
Male, n (%)	109 (77.3)	18 (62.1)	0.130	20 (74.1)	16 (59.3)	0.248
Penetrating injury, n (%)	12 (8.5)	2 (6.9)	0.773	4 (14.8)	2 (7.41)	0.386
Glasgow Coma Scale score, median (IQR)	14 (12–15)	9 (3–13)	0.000	12 (3–14)	9 (3–12.5)	0.445
Systolic blood pressure (mmHg), median (IQR)	84 (70–100)	80 (53–90)	0.051	79 (60–97.5)	77 (46.5–88)	0.228
Injury severity score, median (IQR)	29 (21–42)	38 (33–45)	0.004	35 (25.5–45)	38 (31.5–45)	0.739
Operative time, median (IQR)^a^	144 (96.5–191)	117.5 (93.75–163.5)	0.011	175 (117–214.5)	117.5 (89.25–161.5)	0.011
AIS, n (%)						
AIS head/neck > 3	17 (12.1)	2 (6.9)	0.422	4 (14.8)	2 (7.41)	0.386
AIS chest > 3	31 (22.0)	13 (44.8)	0.001	8 (29.6)	11 (40.7)	0.393
AIS abdomen > 3	116 (82.2)	26 (89.7)	0.329	23 (85.2)	25 (92.6)	0.386
AIS extremity > 3	6 (4.3)	4 (13.8)	0.047	2 (7.4)	4 (14.8)	0.386

*AIS* Abbreviated Injury Scale, *IQR* Interquartile range, *MSL* Multiple-staged laparotomy, *SSL* Single-staged laparotomy

^a^Three cases that underwent laparotomy in the ER did not have their operative time documented

**Table 2:** Dominant sources of hemorrhage of the SSL and MSL group

	Before matching			After matching		
	SSL group n = 141	MSL group n = 29	p value	SSL group n = 27	MSL group n = 27	p value
Hepatic injury, n (%)	54 (38.3)	19 (65.5)	0.007	18 (66.6)	19 (70.4)	0.770
Without juxta-hepatic venous injury	46 (32.6)	13 (44.8)	0.209	14 (51.9)	13 (48.1)	0.785
With juxta-hepatic venous injury	8 (5.7)	6 (20.7)	0.007	4 (14.8)	6 (22.2)	0.484
Splenic injury, n (%)	46 (32.6)	3 (10.3)	0.016	3 (11.1)	3 (11.1)	1.000
Great vessel injury, n (%)	3 (2.1)	3 (10.3)	0.029	3 (11.1)	1 (3.7)	0.299
Pancreas injury, n (%)	7 (5.0)	2 (6.9)	0.672	2 (7.4)	2 (7.4)	1.000
Mesenteric injury, n (%)	29 (20.6)	2 (6.9)	0.083	1 (3.7)	2 (7.4)	0.552

*MSL* Multiple-staged laparotomy, *SSL* Single-staged laparotomy

**Table 3:** Surgical procedures of the SSL group

	Before matching n = 141	After matching n = 27
Hepatectomy, n (%)	28 (19.8)	7 (25.9)
IVC repair, n (%)	10 (7.1)	6 (22.2)
Hepatorrhaphy, n (%)	16 (11.3)	5 (18.5)
Splenectomy, n (%)	32 (22.7)	3 (11.1)
Distal pancreatectomy, n (%)	10 (7.1)	2 (7.4)
Aortic repair, n (%)	1 (0.71)	1 (3.7)
Bowel and mesenteric repair, n (%)	30 (21.3)	1 (3.7)
Splenorrhaphy, n (%)	4 (2.8)	0
Partial splenectomy, n (%)	3 (2.1)	0
Pancreatico-duodenectomy, n (%)	2 (1.4)	0
Others, n (%)	5 (3.5)	2 (7.4)

**Fig. 2:**
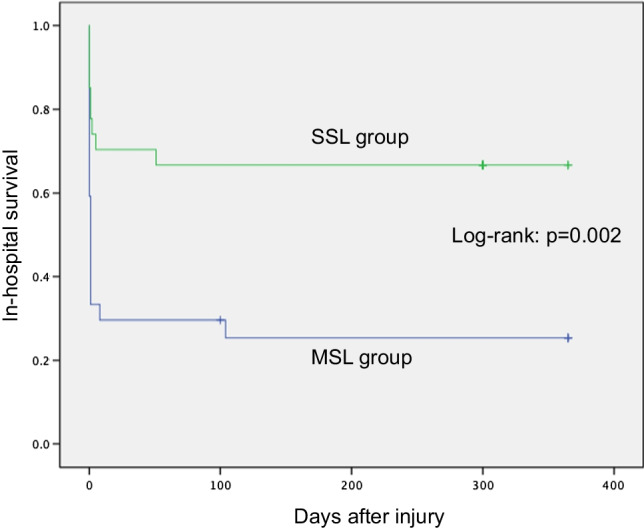
Kaplan–Meier estimation of in-hospital survival in propensity score-matched SSL and MSL groups. The p value was generated with the use of a log-rank test

The in-hospital mortality rate of the entire cohort was 27.6% (47/170), the 48-h mortality rate was 14.7% (25/170), and the 28-day mortality rate was 21.2% (36/170). Table 4 shows in-hospital mortality (primary outcome), 48-h mortality, and 28-day mortality (secondary outcomes), cause of death in the SSL and MSL groups. In the analysis of all patients, in-hospital, 48-day mortality, and 28-day mortality were significantly lower in the SSL group than in the MSL group. The propensity score-matched analysis showed significant differences between the SSL and MSL groups in in-hospital mortality (33.3% vs. 74.1%; odds ratio [OR] 0.154; 95% confidence interval [CI] 0.035–0.682) and 28-day mortality (29.6% vs. 59.3%; HR 0.200; 95% CI 0.044–0.913). But the 48-h mortality did not differ significantly between the two groups (25.9% vs. 44.4%; OR 0.375; 95% CI 0.099–1.414). The 1-year survival curves showed higher survival in the SSL group (log-rank p = 0.002) (Fig. 2).

**Table 4:** Outcomes of SSL and MSL groups

	Before matching		After matching					
	SSL group n = 141	MSL group n = 29	OR	95% CI	SSL group n = 27	MSL group n = 27	OR^a^	95% CI^a^
In-hospital mortality, n (%)	26 (18.1)	21 (72.4)	0.086	0.034–0.216	9 (33.3)	20 (74.1)	0.154	0.035–0.682
48-h mortality, n (%)	12 (8.5)	13 (44.8)	0.114	0.045–0.293	7 (25.9)	12 (44.4)	0.375	0.099–1.414
28-day mortality, n (%)	19 (13.5)	17 (58.6)	0.110	0.045–0.266	8 (29.6)	16 (59.3)	0.200	0.044–0.913
Cause of death, n (%)								
Uncontrollable hemorrhage	11 (7.8)	17 (58.6)	0.060	0.023–0.156	6 (22.2)	16 (59.3)	0.167	0.037–0.745
Multiple organ failure	4 (2.8)	2 (6.9)	0.394	0.069–2.261	3 (11.1)	2 (7.4)	1.500	0.251–8.977
Traumatic brain injury	5 (3.5)	2 (6.9)	0.496	0.091–2.693	0	2 (7.4)	0.015	0.000–1327
Sepsis	2 (1.4)	0			0	0		

*CI* Confidence interval, *MSL* Multiple-staged laparotomy, *OR* odds ratio, *SSL* Single-staged laparotomy

^a^OR and 95% CI were calculated using by conditional logistic regression analyses

## Discussion

The results of this study showed that SSL for traumatic massive hemoperitoneum with hemodynamic instability improved a long-term mortality compared with MSL. Although the Kaplan–Meier graph (Fig. 2) appears to show difference in the very early phase between SSL and MSL groups attributable to increased incidence of bleeding death in MSL group. However, as shown in Table 4, there was no significant difference between the groups for 48-h mortality. Therefore, the difference in the outcomes between the groups could not be attributed to increased incidence of peri-operative bleeding death in MSL group.

Supporting this concept are the data from Harvin et al. showing that DCS was associated with increased mortality [9]. Although Harvin et al. focused on the patients requiring emergent laparotomy for all severe abdominal trauma, the present study may be the first to focus on traumatic massive hemoperitoneum with hemodynamic instability, which most likely leads to DCS.

There were many possible explanations as to why SSL improved a long-term outcome. First, SSL needs only one-time surgery. Patients with severe abdominal injury often have severe concomitant injuries. In particular, a severe brain injury and orthopedic injuries including spinal, pelvic and femoral fractures require both early surgical intervention and multidisciplinary management. A severe head injury requires the head-up position. Early definitive fixations for spinal, pelvic, and femoral fractures are always necessary for early mobilization to prevent pneumonia and venous thromboembolism. Although MSL with an open abdomen and re-operations interfere with the management of these concomitant injuries, primary SSL requiring only one-time surgery can allow us to dedicate subsequent management to concomitant injuries. External fixation in the acute phase of patients undergoing MSL is possible, but fixation is weak, especially in cases of femoral and pelvic fractures. Therefore, early mobilization is difficult with external fixation alone, and we believe that internal fixation as early as possible was associated with reduced complications and improved prognosis. The present data of all patients showed that the death from brain injury, sepsis, or multiple organ injury were rare, which support the association.

Second, hepato-biliary-pancreatic (HBP) surgery has evolved over the decades. Traumatic massive hemoperitoneum is often caused by HBP injuries; therefore, an HBP surgical procedure is often necessary. Currently, establishment of surgical procedures and development of energy devices, surgical staplers, and electrocautery have made hepatectomy and pancreatectomy technically easier and faster. Finally, DCR has evolved and become well known. DCR aims at preventing or reversing coagulopathy through permissive hypotension, limiting crystalloids, and delivering higher ratios of plasma and platelets. Thus, the implementation of DCR allowed us to perform SSL requiring a prolonged surgical procedure. In absolute terms, SSL potentially has a greater hemostatic effect than MSL.

The present in-hospital, 48-h mortality, and 28-day mortality rates of all patients (including cardiac arrest on admission) were 27.6%, 14.7%, and 21.2%, respectively. In 2002, Clarke et al. published data from 1986 to 1999 reporting a mortality rate of 40% for hypotensive trauma patients undergoing laparotomy within 90 min of arrival [7]. In 2017, Harvin et al. reported that the mortality rate of hypotensive trauma patients undergoing emergent laparotomy was 46% [10]. In 2020, Traynor et al. reported that the in-hospital mortality rate of patients who underwent DCS in high-income countries was 29% [11]. Even though these studies excluded patients with cardiac arrest on admission or intra-operative death, the present outcomes including patients with cardiac arrest or intra-operative death were superior to these reports. The present data were satisfactory compared with these other reports, affirming our surgical strategy based on mandatory SSL.

The aim of MSL is to avoid the lethal triad that is described as the metabolic derangement of hypothermia, coagulopathy, and metabolic acidosis [6]. The tenet that the lethal triad leads to a “vicious, bloody cycle” and subsequent irreversible physiological exhaustion is emphasized far too much in abdominal trauma surgery. When the concept of “Damage Control” was first described in 1983 [12], HBP surgery was still a high-risk procedure, and DCR had not yet been established. At that time, expeditious hemostasis with abdominal packing was the only optimal procedure to avoid the lethal triad. However, with current development of HBP surgery and established DCR, MSL is no longer required. Higa et al. reported improved mortality and no abdominal compartment syndrome in severe abdominal trauma requiring laparotomy, despite a decrease in the rate of DCS at 3 years, and they pointed out the overuse of DCS [13].

This study has several limitations. First, the small sample size and single-center design limit the power and generalizability of the present findings. Second, although propensity scores were used to balance the characteristics between the two groups, there could be some other unmeasurable confounders and potential bias. Roberts et al. reported that strong evidence supported metabolic acidosis (with elevated lactate or decreased pH) and hypothermia as indicators of DCS implementation, and weak evidence supported coagulopathy and two injury patterns (Unstable patients with combined abdominal vascular and pancreas gunshot injuries, penetrating trauma requiring over 10 units of packed red blood cells with one or more major vessel injury and two or more visceral injuries) [14]. However, these potential indicators were not included in the propensity score as a cofounder for the following reasons. Indicators of metabolic acidosis such as blood lactate level, pH and base excess, those of coagulopathy such as prothrombin time, activated partial thromboplastin time, fibrinogen level, and d-dimer were frequently not documented in the early cases, with most of them the MSL group. As such, these indicators were excluded because number of the matched cases would be very small if they were included. Additionally, a decrease in blood pH is not indicative of metabolic acidosis in the presence of hypercapnia, as is often the case in the cases. None of the cases enrolled match the two injury patterns mentioned in the report.

In the same contexts, the authors did not include other potential indicators of DCS implementation such as the injury-to-operation time, the amount of intra-operative blood loss, or vital signs during operation in the propensity score for the following reasons. There was a very large variation in time from injury to arrival with some cases receiving treatment before the arrival, whereas the time from arrival to laparotomy was overwhelmingly shorter in the MSL group. As such, the injury-to-laparotomy time was excluded because it do not accurately reflect the physiological status of the cases, and because number of the matched cases would be very small if it was included. The amount of intra-operative blood loss does not accurately reflect the systemic total blood loss as ascites, intestinal juices and other fluids are included and extra-abdominal bleedings such as retroperitoneal bleeding are not counted. Moreover, the total blood loss during the operation increases with prolongation of the operation time. Vital signs during operation are affected by anesthesia and surgical maneuvers such as Pringle’s maneuver.

Finally, the short-term outcome might be largely affected by the attending surgeons. Since there was no clear-cut indication for MSL, attending surgeons had to select the optimal procedures based on each patient’s clinical status, severity of the injured organ, and concomitant injuries. It should be noted that the study is a single center study, where all abdominal trauma cases were attended by surgeons experienced in HBP and other surgeries. This is another limitation of the study, as this is not always the case in other institutions, and the results may not be easily generalizable. Therefore, multi-institutional, prospective, observational studies will be useful in resolving these limitations and remaining questions.

## Conclusions

Single-staged laparotomy may be an effective surgical treatment for the traumatic massive hemoperitoneum cases with hemodynamic instability, if conducted following sufficient damage control resuscitation and performed by an experienced surgeon.

## Supplementary Information

**Additional file 1.** Raw dataset.

## Data Availability

All data generated or analysed during this study are included in this published article and its additional information file.

## Abbreviations

AIS: Abbreviated injury score
CI: Confidence interval
DCR: Damage control resuscitation
DCS: Damage control surgery
GCS: Glasgow Coma Scale
HBP: Hepato-biliary-pancreas
ISS: Injury severity score
IVC: Inferior vena cava
MSL: Multiple-staged laparotomy
OR: Odds ratio
SBP: Systolic blood pressure
SSL: Single-staged laparotomy
